# The probiotic effects of host-associated *Bacillus velezensis* in diets for hybrid yellow catfish (*Pelteobagrus fulvidraco* ♀ × *Pelteobagrus vachelli* ♂)

**DOI:** 10.1016/j.aninu.2023.08.004

**Published:** 2023-08-22

**Authors:** Zhehui Ji, Xing Lu, Mingyang Xue, Yuding Fan, Juan Tian, Lixue Dong, Chuanzhong Zhu, Hua Wen, Ming Jiang

**Affiliations:** a College of Fisheries and Life Science, Shanghai Ocean University, Shanghai, China; b Yangtze River Fisheries Research Institute, Chinese Academy of Fishery Sciences, Wuhan, China; c Fujian Key Laboratory of Functional Aquafeed and Culture Environment Control, Fujian DBN-HY Aquatic Science and Technology Group Co., Ltd, Zhao'an, China

**Keywords:** *Bacillus velezensis*, Probiotics, Hybrid yellow catfish, Intestinal health, Liver metabolome

## Abstract

This study was to evaluate the potential of a host-associated *Bacillus velezensis* as a probiotic for hybrid yellow catfish (*Pelteobagrus fulvidraco ♀* × *Pelteobagrus vachelli* ♂). Diets (B0 to B5) containing 0, 0.90 × 10^8^, 0.80 × 10^9^, 0.85 × 10^10^, 0.90 × 10^11^, 0.83 × 10^12^ CFU/kg *B*. *velezensis* YFI-E109 were fed to the fish with initial weight (3.07 ± 0.08 g) in a recirculating aquaculture system for six weeks with three replicates, respectively. Probiotic effects were analyzed based on growth, body composition, liver and gut morphology, gut microbiome, and liver metabolome. Analysis of the bacterial genome has shown that the most abundant genes in *B*. *velezensis* YFI-E109 were distributed in carbohydrate and amino acid metabolism. Fish in groups B3 and B4 had better growth performance, and higher intestinal amylase (AMS) and lipase (LPS) activities compared with other groups (*P* < 0.05). Fish in groups B0 and B5 showed significant liver damage, while this status improved in group B3. The liver malondialdehyde (MDA) content in group B3 was lower than that in other groups (*P* < 0.05). The abundance of *Mycoplasma, Ralstonia* and *Acinetobacter* was significantly reduced in B3 and B5 compared to B0. The amino acid and carbohydrate metabolism pathways were enriched in group B3 compared with group B0. In conclusion, dietary *B. velezensis* YFI-E109 supplementation has the potential to improve growth, liver metabolism, and liver and gut health, and reshape the gut microbiome of hybrid yellow catfish. Excessive *B. velezensis* YFI-E109 reduced the prebiotic effects. The recommended dietary supplementation of *B. velezensis* YFI-E109 is 0.31 × 10^10^ to 0.77 × 10^11^ CFU/kg for hybrid yellow catfish according to the quadratic regression method by plotting specific growth rate (SGR), feed conversion ratio (FCR), MDA and activities of AMS against dietary *B. velezensis* YFI-E109 levels.

## 1 Introduction

Yellow catfish (*Pelteobagrus fulvidraco*) is an omnivorous freshwater fish native to most rivers, lakes, ditches and other aquatic environments in China. This fish is one of the most popular freshwater aquaculture species in China and the annual output is 565,477 tonnes (MARA, 2022). Hybrid Yellow catfish (*P. fulvidraco ♀* × *Pelteobagrus vachelli ♂*) is one of the main species of yellow catfish used in aquaculture. Intensive pond culture is widely used to meet its market demands considering the increasing production of yellow catfish in China. This model inevitably results in water deterioration due to the ammonification of feed residues and fish excreta, which has produced a mass of ammonia nitrogen resulting in growth reduction, immune suppression, and prolonged stress in fish (Jiang et al., 2021). In intensive aquaculture conditions, yellow catfish are susceptible to bacterial diseases, which may bring serious economic losses (Liu et al., 2021). Consequently, it is necessary to seek effective strategies to improve fish health and prevent diseases.

Probiotics are defined as live microorganisms that can modulate the intestinal microbial balance and provide beneficial effects on the host health by improving growth, metabolism and immune function (El-Saadony et al., 2021). Numerous studies have confirmed that some probiotics could inhibit the adhesion of pathogenic bacteria by acidification of the gut microenvironment through production and accumulation of organic acids or by secretion of antimicrobial metabolites (like bacteriocins) (Yousefi et al., 2019). Simultaneously, probiotics could serve as antagonists of pathogens by competing for limited resources such as nutrients and space (Chauhan and Singh, 2019), or by producing a range of digestive enzymes and nutritional elements (including vitamins or other nutrient outputs) (Tomasik and Tomasik, 2020).

Probiotics have widely used in aquaculture due to their aforementioned biological properties (Dawood et al., 2018). Previous studies have reported that adequate probiotic supplementation in the diet exerts a positive influence on various aquaculture species (Chen et al., 2021b; Zulhisyam et al., 2020; Haque et al., 2021) and has no obvious toxicity to the host animals. Among different probiotic species, *Bacillus velezensis* is regarded as a potential probiotic due to its multiple functions in aquaculture (Khalid et al., 2021). *B. velezensis* has been reported to grow and secrete a wide range of digestive enzymes (including protease, amylase [AMS] or lipase [LPS]) and nutritional metabolites (Wang et al., 2021; Chen et al., 2021a). The literature shows that dietary *B. velezensis* supplementation improves growth of Amur minnow (*Rhynchocypris lagowskii Dybowski*) (Zhang et al., 2022; Chen et al., 2021a). Besides its growth-promoting roles, dietary supplementation with *B*. *velezensis* extracts showed immune stimulating, antimicrobial, and anti-stress effects in Nile tilapia (*Oreochromis niloticus*) (Doan et al., 2018; Kuebutornye et al., 2020a) and European sea bass (Monzón-Atienza et al., 2022). Furthermore, dietary administration of *B. velezensis* also significantly improves the health of the intestinal tract of grass carp (Chang et al., 2022) and reshapes intestinal microbiota in *Litopenaeus vannamei* (Monzón-Atienza et al., 2022). However, its effects and application remain to be further explored in aquaculture.

In our previous work, 65 strains of *Bacillus* were isolated from the intestinal tract of hybrid yellow catfish, and the strain *B. velezensis* YFI-E109 with the strongest antibacterial activity was screened by Oxford cup antagonism test. This strain was considered as a potential probiotic for hybrid yellow catfish. Thus, six test diets contained with different levels of *B. velezensis* YFI-E109 were fed to hybrid yellow catfish for six weeks. Probiotic effects were analyzed based on growth, body composition, liver and gut morphology, gut microbiome, and liver metabolome. The purpose of this study was to determine the probiotics effect and optimal dietary supplemental level of *B. velezensis* YFI-E109 for hybrid yellow catfish.

## 2 Materials and methods

### 2.1 Animal ethics statement

Fish management and sampling protocols were authorized by the Animal Experimental Ethical Inspection of Laboratory Animal Centre, Yangtze River Fisheries Research Institute, Chinese Academy of Fishery Sciences. The protocol number is YFI2021JM01.

### 2.2 *B. velezensis* preparation

The original bacteria were isolated from the midgut of hybrid yellow catfish. The probiotic named *B*. *velezensis* YFI-E109 is stored in Yangtze River Fisheries Research Institute, Chinese Academy of Fishery Sciences (Wuhan, China).

The seed liquid containing *B. velezensis* YFI-E109 for 24 h with an inoculation amount of 2% was injected in the fermentation medium with initial pH of 6.8 prepared as: corn meal (0.5%), soybean meal (1%), sucrose (0.4%), fish meal (0.6%), KH_2_PO_4_ (0.1%), FeSO_4_·7H_2_O (0.025%), MgSO_4_·7H_2_O (0.05%), MnSO_4_ (0.024%), CaCO_3_ (0.1%) and defoamers (0.05%). Fermentation was conducted at 100 revolutions per minute, with a 4:1 VVM (air volume/culture volume per minute) ventilation ratio, and 0.05 N/mm^2^ pressure in the tank. After culture for *B. velezensis* YFI-E109, 15% sterilized brine was supplemented into the medium to stop fermentation. The fermentation solution was centrifuged at 4500 × *g* for 2 min, and the supernatant was removed. PBS solution was added into the sediment and centrifuged for 2 min. This step was repeated three times to obtain *B. velezensis* YFI-E109 solution. The bacteria number of active *B. velezensis* YFI-E109 was measured and diluted with physiological saline solution to 2 × 10^12^ CFU/mL.

### 2.3 Experimental diets

The formulation and proximate composition of the test diets (B0 group) are given in Table 1. Six experimental diets were formulated by adding *B. velezensis* YFI-E109 at the corresponding concentrations of 0, 1 × 10^8^, 1 × 10^9^, 1 × 10^10^, 1 × 10^11^ and 1 × 10^12^ CFU/kg, namely B0 to B5, respectively. All dry ingredients were thoroughly ground using a 0.3-mm size sieve, weighed and mixed with a groove-type mixer (Shanghai Xinxing Food Moulds Co., Ltd, China). The dry mixture was blended for 15 min with fish oil and soybean oil, and the water combined with bacterial diluents was made up to 300 mL/kg diet. Then, the resulting moist mixture was extruded using a meat grinder (TY-432, Shang Hai Tai Yi Machinery, China) with a diameter of 0.8 mm and a length of 0.5 to 1 mm. Meanwhile, a special water condensing tube was utilized to make sure that the temperature was less than 50 °C under the process of feed production. The wet pellets were air-dried in a mesh-belt drier (Changzhou Suzheng Drying equipment Co., Ltd, China) for 4 h at 40 °C. The resulting air-dry pellet diets were saved in plastic bags and stored at −20 °C until use. Diets were defrosted to room temperature before each feeding. The measured concentrations of *B. velezensis* YFI-E109 in the test diets were 0, 0.90 × 10^8^, 0.80 × 10^9^, 0.85 × 10^10^, 0.90 × 10^11^, 0.83 × 10^12^ CFU/kg diet, respectively.

**Table 1:** Dietary formulation and proximate composition of the test diets (%, as air-dried basis).

Ingredients	B0	B1	B2	B3	B4	B5
Fishmeal^1^	30	30	30	30	30	30
Peeling soybean meal^2^	20	20	20	20	20	20
Cottonseed meal^3^	10	10	10	10	10	10
Corn protein powder^4^	10	10	10	10	10	10
Flour^5^	17.2	17.2	17.2	17.2	17.2	17.2
Gluten^6^	2	2	2	2	2	2
Fish oil^1^	3	3	3	3	3	3
Soybean oil^7^	3	3	3	3	3	3
Calcium dihydrogen phosphate	1.5	1.5	1.5	1.5	1.5	1.5
Vitamin premix^8^	1	1	1	1	1	1
Mineral premix^9^	2	2	2	2	2	2
Vitamin C	0.1	0.1	0.1	0.1	0.1	0.1
Choline chloride	0.2	0.2	0.2	0.2	0.2	0.2
*Bacillus velezensis*, CFU/kg	0	1 × 10^8^	1 × 10^9^	1 × 10^10^	1 × 10^11^	1 × 10^12^
Total	100	100	100	100	100	100
Proximate composition (as dry matter basis)						
Crude protein	44.68	46.47	46.01	47.06	46.39	46.21
Crude lipid	9.14	9.44	9.26	9.44	9.24	9.43
Ash	9.26	9.18	9.29	9.26	9.27	9.34

1 Fujian Tianma Technology Group Co., Ltd.

2 Cofco (Dongguan) Grain and Oil Industry Co., Ltd.

3 Xinjiang Jianlan Plant Protein Co., Ltd.

4 Henan Julong Bioengineering Co., Ltd.

5 Yihaijiali (Wuhan) Grain and Oil Industry Co., Ltd.

6 Fengqiu County Huafeng Powder Industry Co., Ltd.

7 Yihaijiali Arowana Food Co., Ltd.

8 The vitamin premix provided the following per kilogram of diets: vitamin A 5,000 IU; vitamin D_3_ 2,000 IU; vitamin E 60 mg; vitamin B_1_ 5 mg; vitamin B_2_ 20 mg; vitamin B_6_ 10 mg; vitamin C 120 mg; vitamin K_3_ 5 mg; inositol 400 mg; nicotinic acid 120 mg; calcium pantothenate 10 mg; folic acid 1 mg; biotin 0.1 mg.

9 The mineral premix provided the following per kilogram of diet: Ca(CH_3_CHOHCOO)_2_ 6540 mg; FeSO_4_ 42.5 mg; MgSO_4_ 1,340 mg; NaH_2_PO_4_ 1,744 mg; NaCl 870 mg; AlCl_3_ 3 mg; KIO_3_ 2.5 mg; KCl 1,500 mg; CuCl_2_ 2 mg; MnSO_4_ 16 mg; CoCl_2_ 20 mg; ZnSO_4_ 60 mg.

### 2.4 Fish maintenance

The hybrid yellow catfish were purchased from Hubei Huangyouyuan Fishery Development Ltd. (Wuhan, China). The fish were acclimatized to an indoor recirculating aquaculture system (RAS) at the Yangtze River Fisheries Research Institute for 2 weeks. The RAS consisted of 18 polycarbonate tanks (500 L each; 400 L of water), a quartz sand cylinder, a denitrification tank, temperature control system and two water pumps. During the period of domestication, the fish were fed the basal diet (B0) twice daily at 3% of their body weight. After acclimation, 450 fish with similar size and no surface injury (mean body weight: 3.07 ± 0.08 g, *n* = 30) were selected and divided into 18 tanks (25 fish per tank) at random. The fish in triplicate tanks were assigned to each dietary treatment. The experimental fish were fed twice a day at 09:00 and 18:00 to apparent satiation for about 30 min each time. The fish in each tank were weighed every 2 weeks to observe growth performance. The mortality was checked daily. The RAS system was maintained every morning before the first feeding. The sand tank that filtered the manure was washed, then about 10% fresh water was added to the RAS to compensate for water loss during system maintenance. Dissolved oxygen and water temperature were tested daily, and additional water quality indicators were tested weekly. During the feeding period, the water temperature was maintained at 27 ± 2.0 °C, pH was 6.8 ± 0.3, dissolved oxygen concentration was 6.05 ± 0.55 mg/L and total ammonia was 0.04 ± 0.01 mg/L, respectively. The feeding trial was maintained under a natural light/dark regime.

### 2.5 Sample collection

Before sampling, all experimental individuals were starved for 24 h. The fish in each tank were batch-weighed and counted for the measurement of weight gain (WG) and survival (SR). Nine fish were collected randomly from each replicate and anaesthetized with a solution of 75 mg/L MS-222 (Sigma, the United States). The condition factor (CF) was obtained from six fish in each replicate tank by calculating the body length and weight. Subsequently, the blood samples were collected from these six fish by puncturing the caudal vein with a 2-mL syringe, clotted at 4 °C for 4 h and centrifuged at 4 °C, 1000 × *g* for 20 min to separate serum for biochemistry analysis. Three fish from each tank were dissected on ice to weigh the viscus and liver tissues, and the viscerosomatic index (VSI) and hepatosomatic index (HSI) were calculated accordingly. After that, the liver and midgut were frozen in liquid nitrogen and then stored at −80 °C to analyze oxidative stress and digestive enzyme activity. Based on the results of growth performance and the principle of minimum trial expenditure, only the frozen liver and midgut in the B0, B3 and B5 groups were selected for further intestinal microflora and liver metabolomics analysis.

### 2.6 Nutritional composition analysis

The (AOAC 2000) method was used to conduct the nutritional composition analysis on both experimental diets and whole fish. In summary, the determination of crude protein was carried out using an automatic Kjelflex nitrogen determination system (Kjelflex K-360; BUCHI Labortechnik AG, Flawil, Switzerland), while crude lipid content was determined through Soxhlet ether extraction. After being placed in a vacuum freeze dryer (Christ Beta 2-4 LD plus LT, Marin Christ Corporation, Osterode, Germany) for 48 h, the samples underwent freeze-drying and were then tested for moisture. Ash content was checked for 24 h at 550 °C using a muffle furnace.

### 2.7 Serum and liver biochemical index analysis

The activities of alanine aminotransferase (ALT), alkaline phosphatase (ALP) and aspartate transaminase (AST), as well as the concentrations of total cholesterol (TCHO), albumin (ALB), total protein (TP), total triglyceride (TG) and glucose (GLU) in serum were determined by an automatic biochemical analyzer (BX-3010, Sysmex Corporation, Tokyo, Japan).

Liver tissues were thoroughly homogenized with 10-fold volumes (w/v) of ice-cold physiological saline solution and centrifuged at 4500 × *g* for 10 min at 4 °C to obtain the supernatant. The level of malondialdehyde (MDA) (Cat. No. A003-1) along with the activities of total superoxide dismutase (SOD) (Cat. No. A003-1), catalase (CAT) (Cat. No. A007-1-1), AMS (Cat. No. C016-1-1) and lysozyme (LYS) (Cat. No. A050-1-1) were analyzed by commercial reagent kits according to the instructions of the manufacturer (Nanjing Jiancheng Bioengineering Institute, Nanjing, China).

### 2.8 Histology analysis

The fixed liver and gut tissues in paraformaldehyde were sent to Wuhan Service Biological Company to make paraffin slices. Tissues were separated with graded ethanol concentrations and washed in xylene, then embedded in paraffin. Microtome sections of 5 mm were cut and stained with hematoxylin and eosin (H&E). A light microscope (Olympus, AX70, Tokyo, Japan) with a camera (Donpisha, XC-003P) was used to observe the slides and Image-Pro Plus 6.0 software (Media Cybernetics, Inc.) was used to analyze electronic images.

### 2.9 Gut microbiota analysis

The gut samples were collected for DNA extraction, and integrity and concentration were measured with 1% agarose gel electrophoresis by a NanoDrop spectrophotometer (ND-1000, DE, USA). An equivalent of 2 ng/μL DNA was used for PCR amplification. The method for 16S rRNA gene amplification has been described in detail in the literature (Yu et al., 2020). Briefly, the primers 338F 5′-ACTCCTACGGGAGGCAGCA-3′ and 806R 5′-GGACTACHVGGGTWTCTAAT-3′ were designed to amplify the V3–V4 regions of the bacterial 16S ribosomal DNA gene. The amplicons were pooled in equimolar solution, gel purified, and then sequenced with paired-ends on an Illumina MiSeq platform. The raw data have been submitted to the NCBI database with the BioProject ID PRJNA900019. Sequence data were demultiplexed, quality filtered, and analyzed with QIIME and R package (v3.2.0). Linear discriminant analysis effect size (LEfSe) was utilized to analyze differentially abundant taxa across groups using the default parameters. Venn analysis was performed to identify the species that were shared and unique across different groups. Nonmetric Multidimensional Scaling analysis (NMDS) was used to analyze the structure of microbial communities in different groups.

### 2.10 Liver metabolome analysis

An internal standard 2-chlorophenylalanine (2.8 mg/mL, 10 L) was added to the fish liver sample (approximately 50 mg). The sample was then ground for 90 s at 65 Hz, followed by vortexing for 30 s and a centrifugation step lasting 15 min (16,102.7 × *g*, 4 °C). The metabolites of the fish sample were separated and detected using UPLC-MS/MS technology. A temperature-controlled column compartment, binary pump, micro degasser, and autosampler were included in the UPLC system. Waters ACQUITY UPLC HSS T3 C18 column separations were carried out on a 1.8-m (2.1 mm × 100 mm) column. The supernatant (200 μL) was analyzed on the UPLC-MS platform, using a binary phase (solvent A, water containing 0.04% formic acid, v/v; solvent B, acetonitrile containing 0.04% formic acid, v/v) elution compound at a flow rate of 0.35 mL/min.

The program for the linear gradient elution utilized was water/formic acid at 95:5 (v/v) for 0 min; 5:95 (v/v) for 11 min; 5:95 (v/v) for 12 min; 95:5 (v/v) for 12.1 min; 95:5 (v/v) for 14 min. The injection volume was 2 mL, and the column was maintained at 40 °C. The separated components were detected with a tandem mass spectrometer (Applied Biosystems 6500 QTRAP, USA) in negative mode. The mass spectrum acquisition conditions were collected as follows: electro spray ionization (ESI) temperature, 120 °C; mass spectrum voltage, 5500 V; curtain gas (CAR), 25 psi; collision-activated dissociation (CAD) parameter set to high. For qualitative analysis, we referred to public mass spectrometry databases such as Wuhan Meiji Biological Company's self-built database, and analyzed the structure of the primary and secondary spectrum data detected by mass spectrometry.

In this experiment, the principal component analysis (PCA) and partial least square discriminant analysis (OPLS-DA) models were used to screen for metabolite differences. The function of differential metabolites, annotation of metabolic networks, and enrichment of pathways were analyzed using the KEGG database.

### 2.11 Statistical analysis

Statistical analyses were performed with SPSS 25.0 (Chicago, the United States). One-way analyses of variance (ANOVA) was performed on the data. Data were presented as mean ± SE (standard error). Tukey's test was used to compare the mean values of treatments when overall differences appeared significant (*P* *<* 0.05). Mass spectrometry data was analyzed using software Analytica 1.6.3.

## 3 Results

### 3.1 *B. velezensis* YFI-E109 genome analysis

**Fig. 1:**
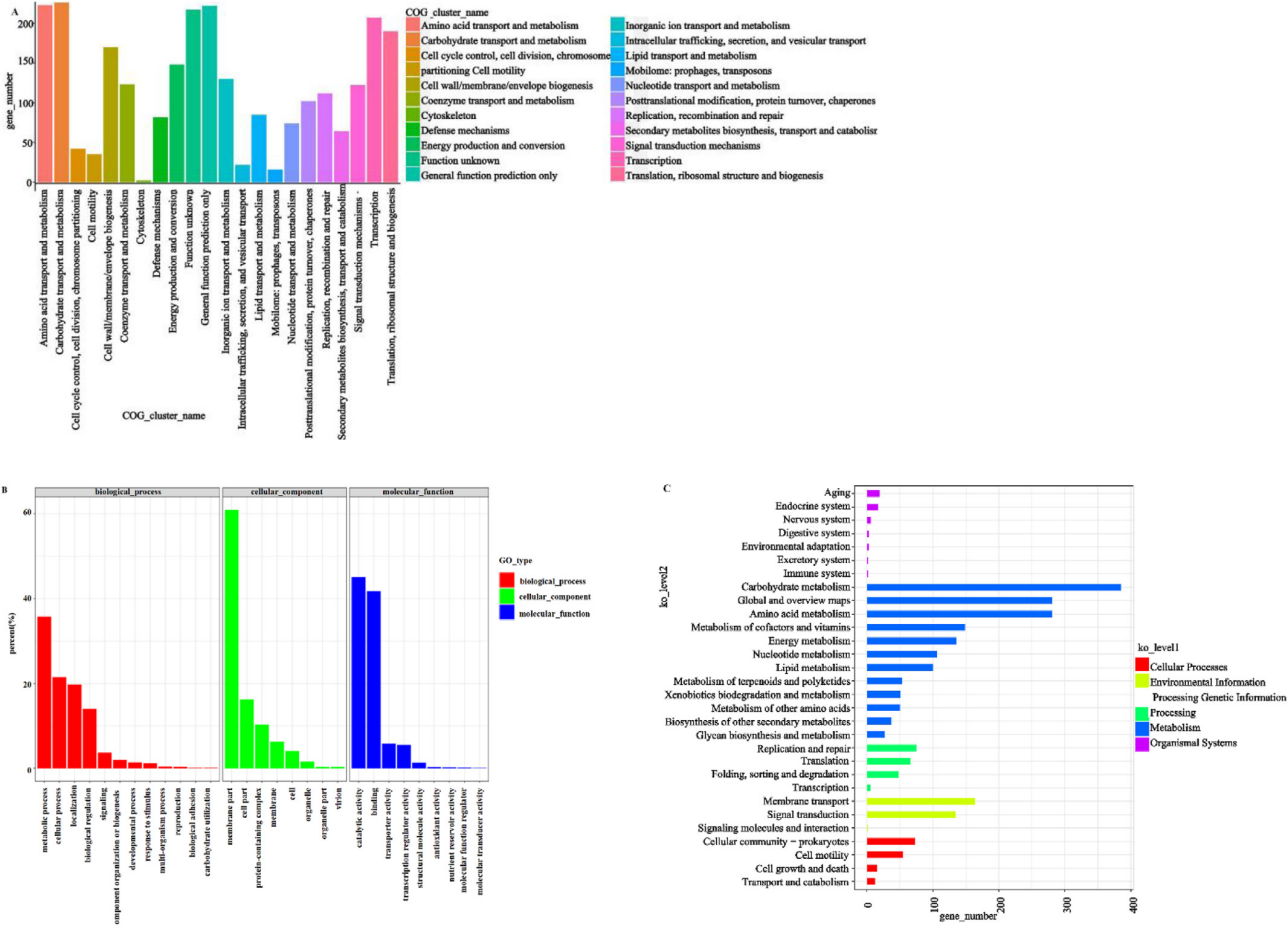
Gene analysis of *Bacillus velezensis* YFI-E109. (A) Clusters of orthologous groups of protein (COG) annotation classification statistics; (B) Gene Ontology (GO) annotation classification statistics; (C) Kyoto encyclopedia of genes and genomes (KEGG) class global and overview maps.

The genome has been submitted to the NCBI database with the BioProject ID PRJNA900000. The coding sequences of *B. velezensis* YFI-E109 were compared for functional clustering as shown in Fig. 1A. The number of genes related to carbohydrate transport and metabolism was the highest in this strain, followed by amino acid transport and metabolism, and major function prediction. The results of GO (Gene Ontology) annotation classification statistics are shown in Fig. 1B. Most of the genes of *B. velezensis* YFI-E109 were related to biological processes. The KEGG class global and overview map (Fig. 1C) showed that most pathways were associated with metabolism. In particular, these metabolic pathways were concerned carbohydrate metabolism and amino acid metabolism.

### 3.2 Growth performance and whole-body composition

**Fig. 2:**
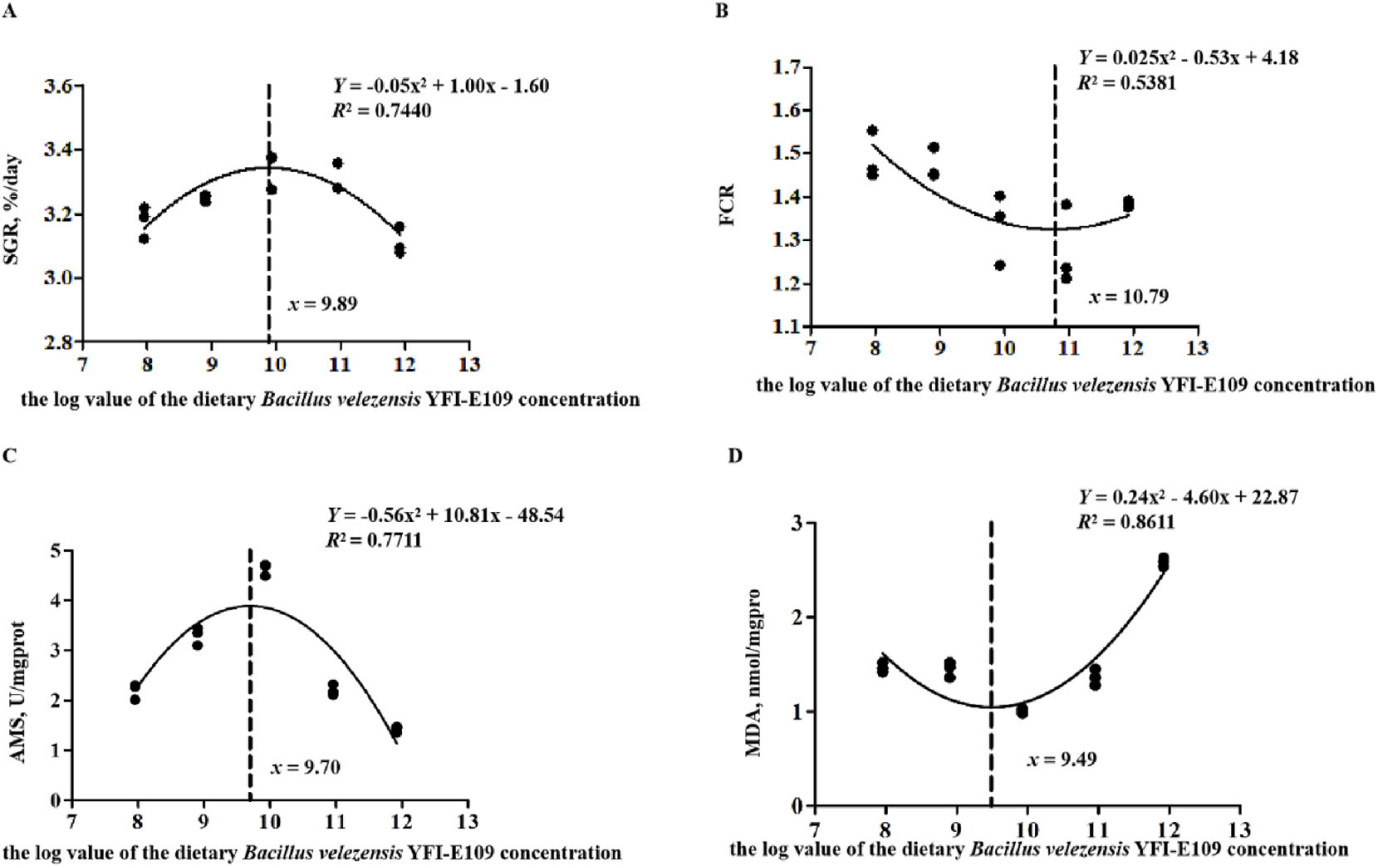
Estimation of the optimal dietary *Bacillus velezensis* YFI-E109 level for hybrid yellow catfish. (A to D) Quadratic regression analysis using the specific growth rate (SGR), feed conversion ratio (FCR), amylase (AMS) and malondialdehyde (MDA). (A) *x* = 9.89 means that the optimum dietary *B. velezensis* YFI-E109 level is 0.77 ✕ 10^10^ CFU/kg. (B) *x* = 10.79 means that the optimum dietary *B. velezensis* YFI-E109 level is 0.77 ✕ 10^11^ CFU/kg. (C) *x* = 9.70 means that the optimum dietary *B. velezensis* YFI-E109 level is 0.50 ✕ 10^10^ CFU/kg. (D) *x* = 9.49 means that the optimum dietary *B. velezensis* YFI-E109 level is 0.31 ✕ 10^10^ CFU/kg. The results were obtained by regression analysis according to the experimental results of five diets containing 0, 0.90 × 10^8^, 0.80 × 10^9^, 0.85 × 10^10^, 0.90 × 10^11^, 0.83 × 10^12^ CFU/kg *Bacillus velezensis* YFI-E109, respectively.

The growth parameters are presented in Table 2. Fish in groups B3 and B4 had excellent performance on WG and specific growth rate (SGR) in comparison with other groups, the lowest value of WG and SGR were observed in group B0 (*P* = 0.000). The opposite trend was shown on feed conversion ratio (FCR), which yielded the lowest value in groups B3 and B4, and the highest value in the B0 group. There was a slight reduction on HSI and VSI in group B4. Dietary *B. Velezensis* YFI-E109 supplementation had no significant impact on CF and SR. The optimum dietary *B. velezensis* YFI-E109 level in hybrid yellow catfish was estimated to be 0.77 ✕ 10^10^ and 0.77 ✕ 10^11^ CFU/kg according to the quadratic regression method by plotting SGR (Fig. 2A) and FCR (Fig. 2B) against dietary *B. velezensis* YFI-E109 levels, respectively.

**Table 2:** Growth performance of hybrid yellow catfish fed test diets supplemented with different levels of *Bacillus velezensis* YFI-E109 for 6 weeks^1^.

Item	B0	B1	B2	B3	B4	B5	ANOVA-*P*
IBW^2^, g	3.06 ± 0.030	3.05 ± 0.052	3.16 ± 0.041	3.05 ± 0.068	3.05 ± 0.061	3.03 ± 0.024	0.489
FBW^3^, g	11.03 ± 0.116^b^	11.59 ± 0.333^ab^	12.38 ± 0.160^a^	12.43 ± 0.211^a^	12.38 ± 0.365^a^	11.21 ± 0.141^b^	0.003
WG^4^, %	260.27 ± 7.277^c^	279.85 ± 4.609^bc^	291.36 ± 1.024^ab^	307.06 ± 5.910^a^	305.35 ± 4.251^a^	269.3 ± 3.802^bc^	0.000
SGR^5^, %/d	3.05 ± 0.048^c^	3.18 ± 0.029^bc^	3.25 ± 0.006^ab^	3.34 ± 0.033^a^	3.33 ± 0.026^a^	3.11 ± 0.025^bc^	0.000
FCR^6^	1.52 ± 0.046^a^	1.49 ± 0.032^ab^	1.47 ± 0.021^ab^	1.33 ± 0.047^bc^	1.28 ± 0.053^c^	1.38 ± 0.004^abc^	0.004
HSI^7^, %	1.62 ± 0.152	1.51 ± 0.044	1.35 ± 0.038	1.60 ± 0.110	1.49 ± 0.100	1.58 ± 0.104	0.452
VSI^8^, %	6.49 ± 0.249^a^	5.91 ± 0.136^ab^	5.33 ± 0.165^b^	6.47 ± 0.330^a^	5.34 ± 0.302^b^	6.32 ± 0.370^a^	0.024
CF^9^, g/cm^3^	1.84 ± 0.036	1.83 ± 0.036	1.64 ± 0.024	1.69 ± 0.040	1.87 ± 0.048	1.73 ± 0.095	0.058
SR^10^, %	92.00 ± 2.309	93.44 ± 3.509	86.67 ± 4.807	90.67 ± 3.527	96.00 ± 4.000	96.00 ± 4.000	0.519

IBW = initial mean body weight; FBW = final mean body weight; WG = percentage of weight gain; SGR = specific growth rate; FCR = feed conversion ratio; HSI = hepatosomatic index; VSI = viscerosomatic index; CF = condition factor; SR = survival.

Data are presented as mean ± SE, *n* = 3.

^a, b, c^ Means in the same line sharing the same superscript letter are not significantly different, as determined by Tukey's test (*P* > 0.05).

1 The diets B0, B1, B2, B3, B4 and B5 contained 0, 0.90 × 10^8^, 0.80 × 10^9^, 0.85 × 10^10^, 0.90 × 10^11^, 0.83 × 10^12^ CFU/kg *Bacillus velezensis* YFI-E109, respectively.

2 IBW = initial total fish weight per tank/initial fish number per tank.

3 FBW = final total fish weight per tank/final fish number per tank.

4 WG = 100(FBW–IBW)/IBW.

5 SGR = 100 [(ln final body weight − ln initial body weight)/days].

6 FCR = feed intake per tank/(total final fish weight – total initial fish weight + dead fish); FCR was calculated by dry matter.

7 HSI = 100 (liver weight/body weight).

8 VSI = 100 (viscera weight/body weight).

9 CF = 100 [body weight/(body length)^3^].

10 SR = 100 (final fish number/initial fish number).

The crude protein and lipid content of whole fish displayed an upward trend from group B0 to B5 (Table 3). Fish in group B5 had the highest crude protein content, while the highest value for crude lipid was found in group B4. The lowest ash content was shown in group B1.

**Table 3:** Proximate composition of hybrid yellow catfish fed test diets supplemented with different levels of *Bacillus velezensis* YFI-E109 for 6 weeks^1^.

Item	B0	B1	B2	B3	B4	B5	ANOVA-*P*
Moisture, %	79.54 ± 0.709	77.83 ± 0.605	77.98 ± 0.494	77.29 ± 1.320	77.33 ± 0.531	76.85 ± 0.387	0.226
Crude protein, %	12.02 ± 0.291^c^	12.33 ± 0.100^bc^	12.70 ± 0.183^abc^	12.96 ± 0.035^abc^	13.06 ± 0.237^ab^	13.54 ± 0.244^a^	0.002
Crude lipid, %	5.61 ± 0.026^e^	6.16 ± 0.031^d^	6.30 ± 0.040^c^	6.44 ± 0.016^b^	7.05 ± 0.033^a^	6.03 ± 0.010^d^	0.000
Ash, %	2.85 ± 0.065^a^	2.53 ± 0.044^b^	2.92 ± 0.040^a^	2.95 ± 0.068^a^	2.93 ± 0.035^a^	2.94 ± 0.040^a^	0.000

Data are presented as mean ± SE, *n* = 3.

^a, b, c^^, d, e^ Means in the same line sharing the same superscript letter are not significantly different, as determined by Tukey's test (*P* > 0.05).

1 The diets B0, B1, B2, B3, B4 and B5 contained 0, 0.90 × 10^8^,0.80 × 10^9^, 0.85 × 10^10^, 0.90 × 10^11^, 0.83 × 10^12^ CFU/kg *Bacillus velezensis* YFI-E109, respectively.

### 3.3 Serum biochemical indices

The serum biochemical indices are shown in Table 4. Dietary *B. velezensis* YFI-E109 supplementation significantly decreased the serum TG content (*P* = 0.002), and the highest value of TG was detected in group B0. Moreover, fish in group B3 displayed the highest value of TP and ALB. The fish in group B5 had the highest activity of AST and lowest content of GLU among the groups.

**Table 4:** Serum biochemical indices of hybrid yellow catfish fed diets supplemented with different levels of *Bacillus velezensis* YFI-E109 for 6 weeks^1^.

Item	B0	B1	B2	B3	B4	B5	ANOVA-*P*
TP, g/L	26.36 ± 0.396^b^	28.53 ± 0.902^ab^	33.30 ± 0.227^a^	33.59 ± 0.574^a^	30.28 ± 1.036^a^^b^	27.78 ± 0.583^ab^	0.000
ALB, g/L	6.49 ± 0.035^b^	7.13 ± 0.254^ab^	8.24 ± 0.679^a^	8.10 ± 0.277^a^	7.21 ± 0.266^ab^	6.94 ± 0.143^ab^	0.022
AST, U/L	617.33 ± 31.104^ab^	523.33 ± 32.028^b^	609.33 ± 9.939^ab^	616.67 ± 26.034^ab^	679.00 ± 8.7^a^	656.83 ± 7.3^a^	0.006
ALT, U/L	75.33 ± 10.729	68.767 ± 14.438	56.67 ± 9.333	92.50 ± 5.48	69.50 ± 16.454	72.67 ± 5.044	0.405
ALP, U/L	25.00 ± 3.215	28.67 ± 6.438	32.00 ± 4.163	35.50 ± 2.598	29.00 ± 2.309	24.17 ± 2.205	0.340
GLU, mmol/L	5.62 ± 0.148^a^	5.02 ± 0.244^a^	5.15 ± 0.073^a^	5.37 ± 0.186^a^	3.27 ± 0.574^b^	2.36 ± 0.345^b^	0.000
TG, g/L	5.40 ± 0.403^a^	2.59 ± 0.337^b^	3.32 ± 0.273^b^	3.82 ± 0.033^ab^	3.43 ± 0.517^b^	3.19 ± 0.118^b^	0.002
TCHO, mmol/L	3.13 ± 0.091	3.27 ± 0.280	3.92 ± 0.017	3.37 ± 0.242	3.61 ± 0.214	2.98 ± 0.448	0.242

TP = total protein; ALB = albumin; AST = aspartate aminotransferase; ALT = alanine aminotransferase; ALP = alkaline phosphatase; GLU = glucose; TG = total triglyceride; TCHO = total cholesterol.

Data are presented as mean ± SE, *n* = 3.

^a, b, c^ Means in the same line sharing the same superscript letter are not significantly different, as determined by Tukey's test (*P* > 0.05).

1 The diets B0, B1, B2, B3, B4 and B5 contained 0, 0.90 × 10^8^, 0.80 × 10^9^, 0.85 × 10^10^, 0.90 × 10^11^, 0.83 × 10^12^ CFU/kg *Bacillus velezensis* YFI-E109, respectively.

### 3.4 Oxidative stress and digestive enzyme activity analysis

As shown in Table 5, the SOD activity and MDA content of liver in group B3 were significantly lower than those in other groups (*P* = 0.000), which first decreased and then tended to grow with the increase of *B. velezensis* level. Fish in group B1 and B2 had the highest CAT activity in liver tissues (*P =* 0.000). The optimum dietary *B. velezensis* YFI-E109 level in hybrid yellow catfish was estimated to be 0.31 ✕ 10^10^ CFU/kg according to the quadratic regression method by plotting MDA (Fig. 2D) against dietary *B. velezensis* YFI-E109 levels.

**Table 5:** Liver oxidative stress and gut digestive enzyme activity of hybrid yellow catfish fed diets supplemented with different levels of *Bacillus velezensis* YFI-E109 for 6 weeks^1^.

Item	B0	B1	B2	B3	B4	B5	ANOVA-*P*
MDA, nmol/mgprot	1.46 ± 0.023^b^	1.47 ± 0.029^b^	1.45 ± 0.047^b^	1.00 ± 0.015^c^	1.36 ± 0.049^b^	2.59 ± 0.026^a^	0.000
SOD, U/mgprot	679.88 ± 4.76^a^	465.98 ± 16.030^c^	442.84 ± 5.142^c^	441.00 ± 16.011^c^	441.09 ± 7.307^c^	533.10 ± 7.811^b^	0.000
CAT, U/mgprot	49.35 ± 1.626^b^	61.59 ± 0.756^a^	62.66 ± 0.642^a^	47.16 ± 0.576^b^	45.75 ± 0.546^b^	47.06 ± 0.483^b^	0.000
AMS, U/mgprot	1.74 ± 0.012^d^	2.19 ± 0.091^c^	3.30 ± 0.102^b^	4.63 ± 0.069^a^	2.20 ± 0.062^c^	1.42 ± 0.035^d^	0.000
LPS, U/gprot	22.35 ± 1.059^b^	24.56 ± 0.563^b^	24.29 ± 0.180^b^	33.39 ± 0.521^a^	35.6 ± 0.271^a^	11.03 ± 0.382^c^	0.000

MDA = malondialdehyde; SOD = superoxide dismutase; CAT = catalase; AMS = amylase; LPS = lipase.

Data are presented as mean ± SE, *n* = 3.

^a, b, c^^, d^ Means in the same line sharing the same superscript letter are not significantly different, as determined by Tukey's test (*P* > 0.05).

1 The diets B0, B1, B2, B3, B4 and B5 contained 0, 0.90 × 10^8^, 0.80 × 10^9^, 0.85 × 10^10^, 0.90 × 10^11^, 0.83 × 10^12^ CFU/kg *Bacillus velezensis* YFI-E109, respectively.

The activities of AMS and LPS in the intestine significantly increased but then decreased with increasing *B. velezensis* YFI-E109 level, and the highest activities were observed in groups B3, respectively (*P =* 0.000). The activities of AMS and LPS in group B5 were lower than others. The optimum dietary *B. velezensis* YFI-E109 level in hybrid yellow catfish was estimated to be 0.50 ✕ 10^10^ CFU/kg according to the quadratic regression method by plotting AMS (Fig. 2C) against dietary *B. velezensis* YFI-E109 levels.

### 3.5 Liver and intestinal morphology

**Fig. 3:**
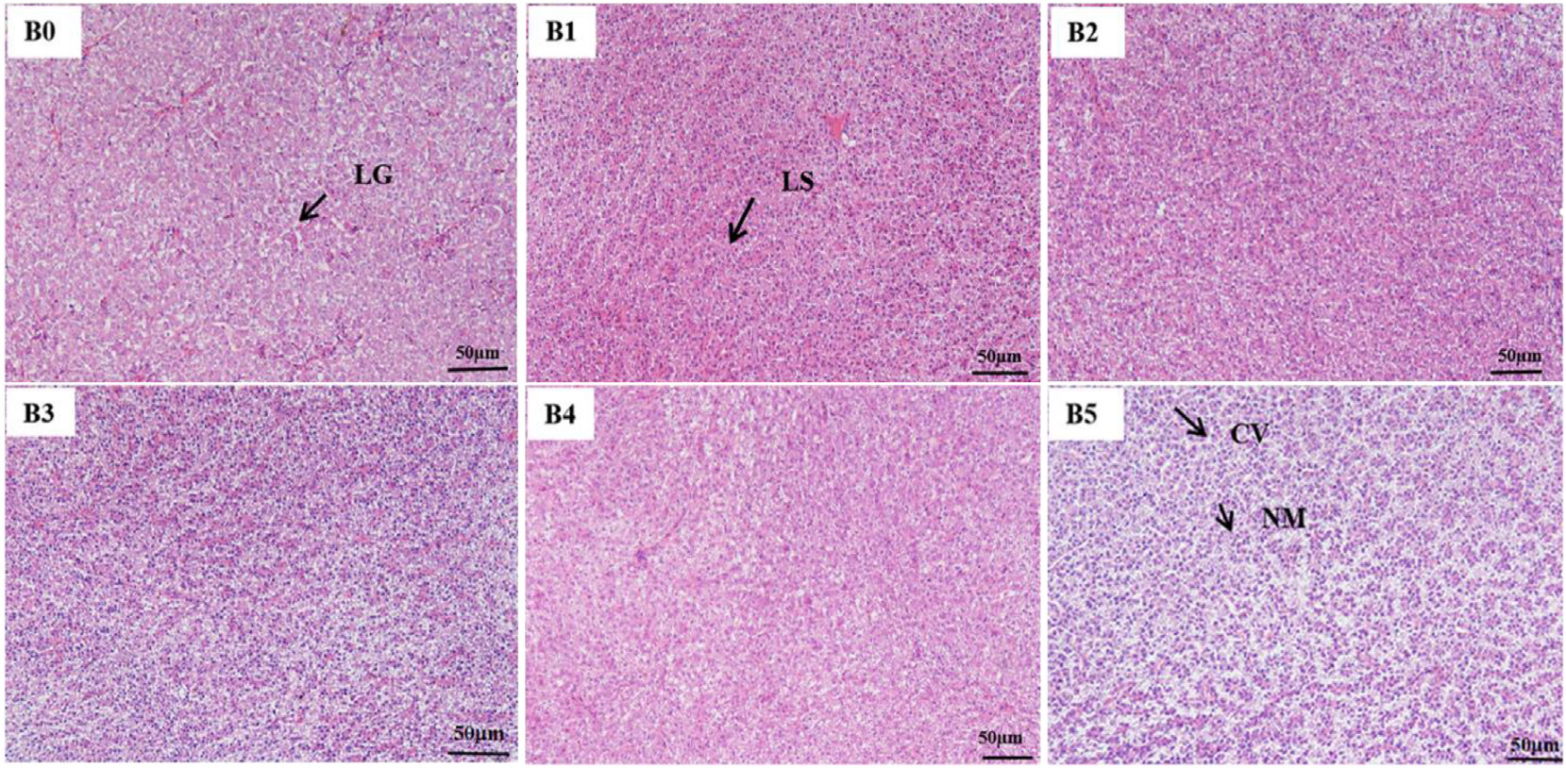
Histological liver sections of hybrid yellow catfish fed with six diets (200× , scale bar = 50 μm). LS = hepatocyte swelling; NM = nuclear migration; LG = intercellular gap; CV = cellular vacuoles.The diets B0, B1, B2, B3, B4 and B5 contained 0, 0.90 × 10^8^, 0.80 × 10^9^, 0.85 × 10^10^, 0.90 × 10^11^, 0.83 × 10^12^ CFU/kg *Bacillus velezensis* YFI-E109, respectively.

**Fig. 4:**
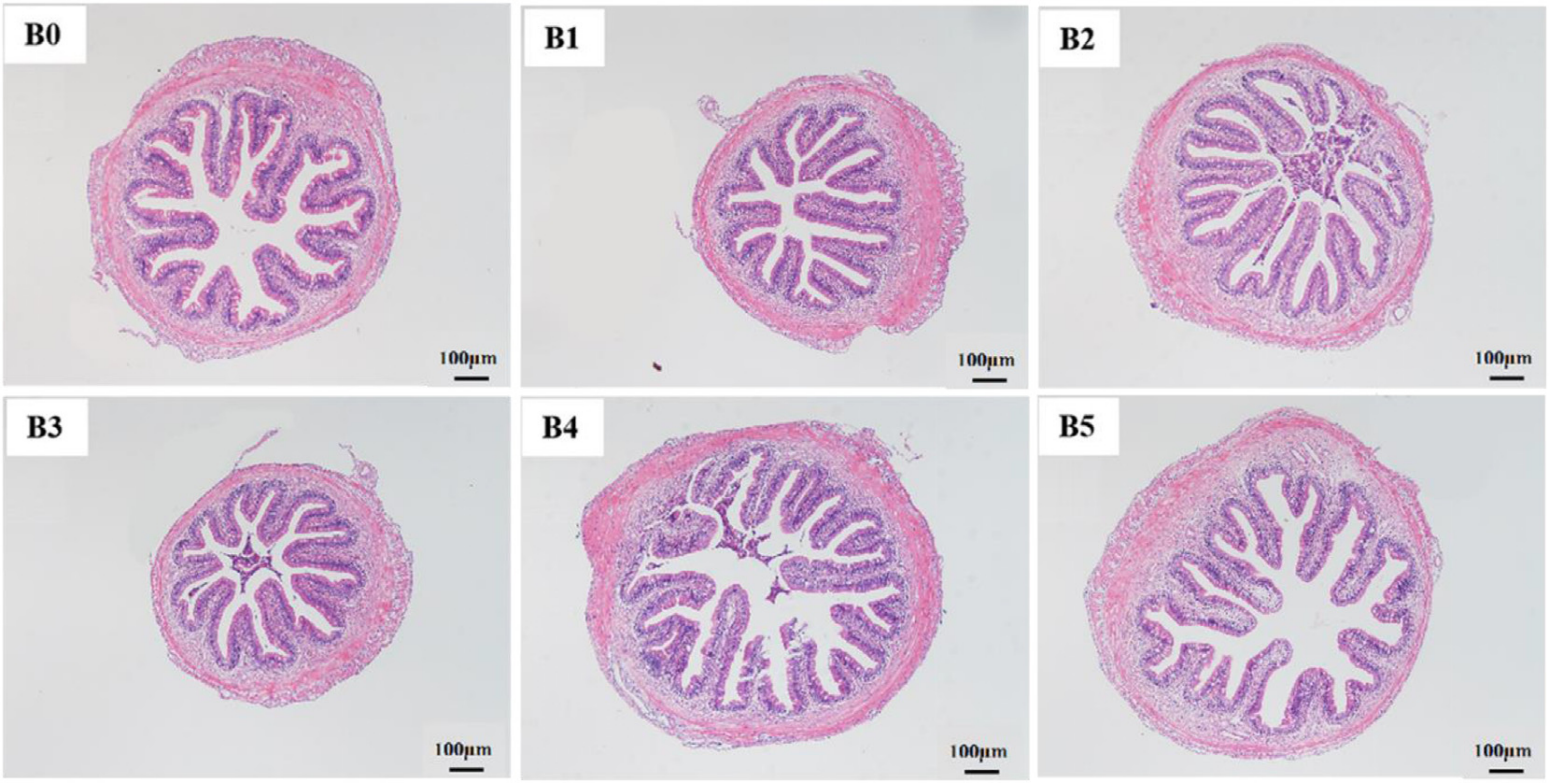
Histological midgut sections of yellow catfish fed with six diets (100× , scale bar = 100 μm). The diets B0, B1, B2, B3, B4 and B5 contained 0, 0.90 × 10^8^, 0.80 × 10^9^, 0.85 × 10^10^, 0.90 × 10^11^, 0.83 × 10^12^ CFU/kg *Bacillus velezensis* YFI-E109, respectively.

The H&E staining sections in liver and intestine are listed in Fig. 3, Fig. 4. Fish in groups B0 and B5 showed obvious liver damage with serious nuclear migration, hepatocyte vacuolization and augmentation of cell gap. This unhealthy liver status was clearly improved in group B3. Supplementary Table S1 shows the values of villus height, villus width, muscularis thickness and intestinal inner diameter of intestinal slices across the six groups. Compared to group B0, the muscularis thickness in group B5 was distinctly increased, and the villus width in group B2 was obviously increased.

### 3.6 Microbial diversity analysis

The Venn diagram demonstrating the distribution of OTUs shared is shown in Supplementary Fig. S1. After regression with 97% similarity of the high-quality sequences, 354, 165 and 131 operational taxonomic units (OTUs) were obtained from groups B0, B3 and B5, respectively, and groups B0, B3 and B5 obtained 263, 91, and 55 unique OTUs, respectively. Supplementary Table S2 shows the alpha diversity analysis in these three groups, where fish had the lowest Chao and ACE value in group B3. In comparison with group B0, fish fed the *B. velezensis* YFI-E109 diet decreased Chao and Simpson index but showed no significant differences in each group. The phylum heatmap in Supplementary Fig. S2 shows a clear distinction in the gut microbiota structure between group B0 and other groups.

**Fig. 5:**
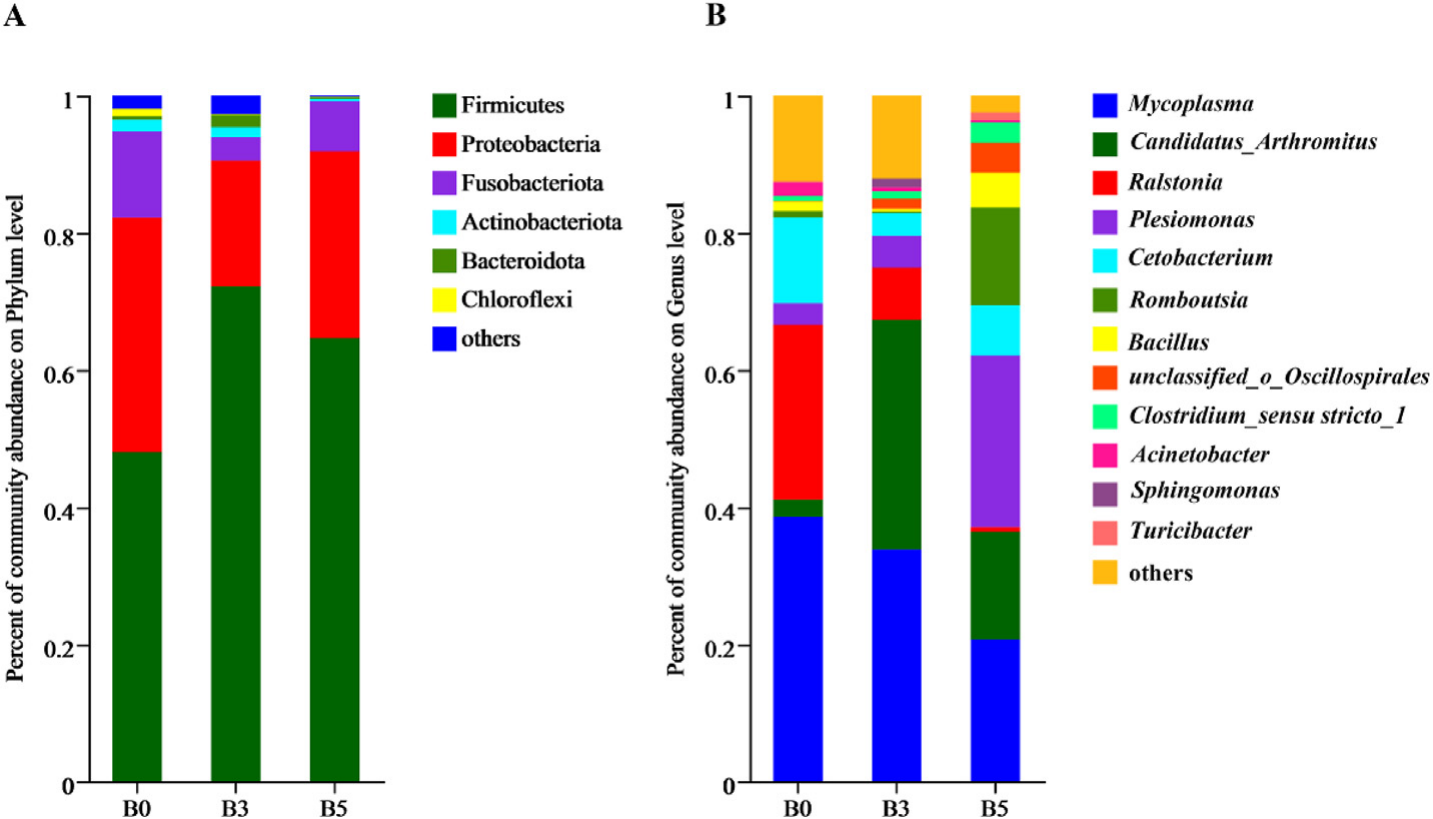
Relative read abundance of different bacteria within the different communities on levels of phylum (A) and genus (B). The diets B0, B3, and B5 contained 0, 0.85 × 10^10^, 0.83 × 10^12^ CFU/kg *Bacillus velezensis* YFI-E109, respectively.

Firmicutes*,* Proteobacteria and Fusobacteria were the predominant phyla in all groups (Fig. 5A) at the phylum level. The Firmicutes abundance in both groups B3 and B5 was markedly increased, while the abundance of Proteobacteria and Fusobacteria decreased compared to group B0. The gut microbiota was dominated by *Mycoplasma*, *Candidatus Arthromitus, Ralstonia* and *Plesiomonas* at the genus level (Fig. 5B). The highest and lowest abundance of *Candidatus Arthromitus* was found in groups B3 and B0, respectively. Compared to group B0, the abundance of *Mycoplasma, Ralstonia* and *Acinetobacter* significantly decreased in both groups B3 and B5. The abundance of *Plesiomonas, Romboutsia* and *Bacillus* was significantly higher in group B5 than other groups.

### 3.7 Liver metabolomic analysis

Liver samples from groups B0, B3 and B5 were chosen for the metabolic analysis. As shown in Supplementary Fig. S3, the orthogonal partial least squares discriminant analysis (OPLS-DA) was performed to exclude the influence of individual differences on metabolism. In the positive and negative ion-modes, B3 vs. B0 and B5 vs. B0 were both distinguished and the samples in same group are clustered together. The different metabolic patterns between every comparison were recognized according to their VIP > 1 and *P* < 0.05 values. As shown in Supplementary Fig. S4, the metabolic numbers for two different comparisons (B3 vs. B0, B5 vs. B0, and B5 vs. B3) were 28 (17 in the positive mode and 11 in the negative mode), 62 (34 in the positive mode and 28 in the negative mode) and 65 (38 in the positive mode and 27 in the negative mode) of various metabolites, respectively.

Using the fold change value as a standard, these differential metabolites were sorted by multiple difference and the major differential metabolites are listed in Supplementary Table S3. Compared with the B0 group, 3,4-dimethylbenzoic acid, N-(1-deoxy-1-fructosyl) phenylalanine, 5-aminovaleric acid, cyclo (Phe–Glu) etc., were significantly up-regulated, while uridine diphosphate glucose (UDPG), UDP-D-galactose, N-acetyl-L-glutamic acid, trigonelline etc., were down-regulated.

**Fig. 6:**
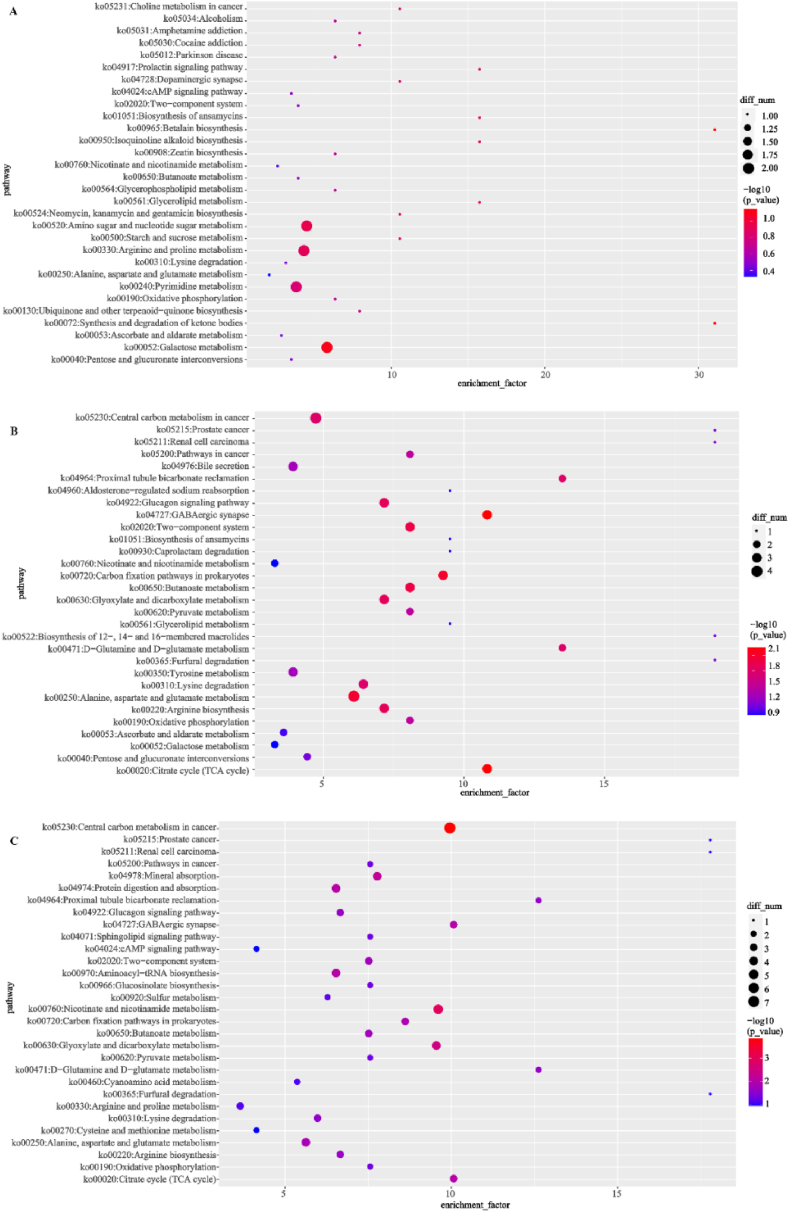
Bubble plot analysis of KEGG enrichment of differential metabolites based on integrated mode. (A) B3 vs. B0; (B) B5 vs. B0; (C) B5 vs. B3. The diets B0, B3, and B5 contained 0, 0.85 × 10^10^, 0.83 × 10^12^ CFU/kg *Bacillus velezensis* YFI-E109, respectively.

As depicted in Fig. 6, KEGG pathway enrichment analysis revealed the top 30 pathways in each group comparison. Among them, most metabolic pathways belonged to metabolism and organismal systems. In B3 vs. B0, the differential metabolites were enriched in amino acid metabolism (include amino sugar and nucleotide sugar metabolism, arginine and proline metabolism), carbohydrate metabolism (galactose metabolism) and nucleotide metabolism (pyrimidine metabolism). Notably, the most highly enriched pathways in group B3 were lipid metabolism (synthesis and degradation of ketone bodies) and amino acid metabolism (betalain biosynthesis). In B5 vs. B0 group, the differential metabolites were enriched in nerve systems Gamma-aminobutyric acid ergic synapse (GABAergic synapse), carbohydrate metabolism (butanoate metabolism, citrate cycle (TCA cycle), carbon fixation pathways in prokaryotes, butanoate metabolism, glyoxylate and dicarboxylate metabolism, proximal tubule bicarbonate reclamation, pyruvate metabolism, pentose and glucuronate interconversions, ascorbate and aldarate metabolism, galactose metabolism), amino acid metabolism (alanine, aspartate and glutamate metabolism, and arginine biosynthesis etc.

## 4 Discussion

The physiological status of fish is often measured by growth performance and whole-body composition. In this study, addition of *B. velezensis* YFI-E109 at a dosage of 0.80 × 10^9^ (B2), 0.85 × 10^10^ (B3) and 0.9 × 10^11^ CFU/kg (B4) significantly increased WG with decreasing FCR in hybrid yellow catfish. This may be related to the genetic composition of *B. velezensis* YFI-E109. Most genes in *B. velezensis* YFI-E109 were enriched in pathways related to metabolic regulation, and most of the proteins encoded by the genome were also involved in carbohydrate transport and metabolism, amino acid transport and metabolism. This suggests that *B. velezensis* YFI-E109 may have a strong ability to metabolize carbohydrates and proteins, thereby facilitating the decomposition and absorption of nutrients in hybrid yellow catfish, thus having the effect of promoting growth. Moreover, compared with others, the content of crude protein and crude lipid in whole fish were increased in diets B2, B3 and B4 that contained 0.80 × 10^9^, 0.85 × 10^10^ and 0.90 × 10^11^ CFU/kg *B. velezensis* YFI-E109, respectively. These results could be explained by the fact that *B. velezensis* can produce various digestive enzymes (Adeniji et al., 2019) to facilitate the decomposition and aggradation of protein and lipid. However, excessive doses of *B. velezensis* YFI-E109 may reduce these beneficial effects in this species of fish. Similar results have previously been reported in the literature (Akter et al., 2019). It is probable that *B. velezensis* YFI-E109 may produce excessive enzymes that compete for endogenous enzymes, resulting in increased deposition of nutrients during the metabolism stage. Moreover, a mass of bacteria colonizing the fish gut may destroy the micro-ecological environment and induce intestinal inflammation, which may slow fish growth (Cerezuela et al., 2013). Similarly, H&E-stained sections may indicate intestinal inflammation in group B5.

The alteration of serum biochemical parameters can indicate the nutritional status of fish (Shoaib et al., 2016). Commonly, the TG level in serum is positively correlated with lipid metabolism (Hokanson, 1998). From our data, dietary *B. velezensis* YFI-E109 administration significantly reduced serum TG levels, suggesting that dietary *B. velezensis* YFI-E109 may enhance lipid utilization and thus reduce lipid deposition in fish. This result is consistent with the findings in zebrafish (Zhang et al., 2023). Data analysis of liver metabolism showed that the most enriched pathway in the B3 group was lipid metabolism, which also directly confirmed this result. ALB, as the main component of TP, is produced in the liver and has vital roles in lipid metabolism, osmotic balance, and transport of metabolic substances (Lermen et al., 2004). In the current study, both serum ALB and TP levels in groups B2, B3 and B4 were increased by the inclusion of *B. velezensis* YFI-E109 in the diet, demonstrating that dietary *B. velezensis* YFI-E109 could regulate lipid metabolism in this fish.

Glucose is regarded as a crucial component of cells and serves as the main energy source utilized by fish utilization (Sun et al., 2021). Under normal physiological conditions, the decrease in glucose levels is in response to increased anabolism in the body. In the current study, serum glucose levels in groups B4 and B5 were significantly lower than those in group B0. KEGG analysis of the bacterial genome revealed that carbohydrate metabolism was the most enriched pathway. In addition, KEGG pathway enrichment analysis in liver metabolome also indicated that carbohydrate metabolism was a major differential metabolic pathway. All of the above information suggests that dietary *B. velezensis* YFI-E109 has a strong regulatory effect on carbohydrate metabolism of hybrid yellow catfish, resulting in a reduction in serum glucose. The decrease in serum glucose indicates that dietary supplementation of *B. velezensis* YFI-E109 with appropriate dosage could elevate anabolism in hybrid yellow catfish. This illustrates that *B. velezensis* YFI-E109 in the diet improves the growth of hybrid yellow catfish.

MDA is known to indicate the lipid peroxidation rate or reflect tissue peroxidation damage and CAT is the marker enzyme of the peroxisome (Monzón-Atienza1 et al., 2022). In our trial, a certain amount of *B. velezensis* YFI-E109 was added to the feed, increasing the activity of CAT in the liver and reducing the content of MDA. But when supplementation was raised to 0.83 × 10^12^ CFU/kg, the body's antioxidant capacity decreased, MDA accumulation increased, and CAT activity decreased sharply.

The gut health of fish is directly associated with nutrient utilization. Intestinal villi are the main location for nutrient digestion and absorption, and the muscle layer is principally related to intestinal contraction force and peristalsis (Smirnov et al., 2004). In our experiment, dietary *B. velezensis* YFI-E109 supplementation with 0.80 × 10^9^ CFU/kg significantly increased the width of intestinal villi in hybrid yellow catfish, inferring increased interaction of the small intestine with nutrients, thereby enhancing absorptive capacity and efficiency (Dawood et al., 2020). Moreover, *B. velezensis* YFI-E109 also increased the muscle layer thickness in this fish species, suggesting a reduction in mechanical damage caused by feed and acceleration in intestinal peristalsis to consume nutrients (Dawood, 2020). Nevertheless, excessive thickness of the muscle layer would lead to incomplete absorption of nutrients (Liu et al., 2018). This may illustrate that the amount of *B. velezensis* YFI-E109 with 0.83 × 10^12^ CFU/kg decreases the weight gain of hybrid yellow catfish. Previous reports have demonstrated that dietary probiotic supplementation could cause physiological changes in the intestinal environment (Zhu et al., 2021; Kuebutornye et al., 2020b). In the present study, intestinal villi damage and proliferation occurred in the intestines of fish fed the 0.83 × 10^12^ CFU/kg *B. velezensis* YFI-E109 diet, whereas the intestinal lesions were remarkably ameliorated in fish fed the diet with 0.80 × 10^9^ and 0.85 × 10^10^ CFU/kg *B. velezensis* YFI-E109. These results indicated that appropriate dietary *B. velezensis* YFI-E109 supplementation may improve the intestinal health of yellow catfish in laboratory conditions, but overdose can damage the fish's gut morphology. Similar observations have been reported in Nile tilapia (Kuebutornye et al., 2020b).

Many studies have shown that the structural alterations in fish intestine may be related to secondary metabolites or other bioactive substances that secreted by gut microbes (Merrifield et al., 2009; Sáenz de Rodrigáñez et al., 2009). This balance between the host and gut microorganisms (including its composition and abundance) is of vital importance to maintain normal gut function in fish (Ley et al., 2008; Zilber-Rosenberg and Rosenberg, 2008). In many studies, increased Proteobacteria abundance was associated with many diseases or inflammatory reactions (Shin et al., 2015) such as obesity, decreased metabolism (Fei and Zhao, 2013), epithelial immune dysfunction (Litvak et al., 2017), and enteritis (Kau et al., 2015). Shin et al. (2015) proposed that an increased abundance of Proteobacteria in the intestine could be used as a marker of intestinal flora imbalance. In addition, the abundance of Firmicutes is positively correlated with animal body fatness (Bradlow, 2014; Turnbaugh et al., 2006). Polysaccharide hydrolase produced by Firmicutes in the gut can effectively decompose dietary fiber to produce monosaccharides and short-chain fatty acids, and is an energy source for the basic metabolism of intestinal cells (Mountfort et al., 2002). In this study, the diets supplemented with *B. velezensis* YFI-E109 at 0.85 × 10^10^ and 0.83 × 10^12^ CFU/kg significantly decreased the richness of Proteobacteria, *Acinetobacter* and *Mycoplasma*, but increased the abundance of Firmicutes, *Arthromitus* and *Bacillus*. These appear to be signs of improved gut microbiome health. Similar results were reported by Kormas et al. (2014). It is shown that Proteobacteria and Actinobacteriota are closely associated with intestinal inflammation and immune disorders, and the increase in their relative abundance may due to an imbalance in intestinal microbial communities (Mohapatra et al., 2012). Semova et al. (2012) indicated that Firmicutes plays important roles in promoting the absorption of intestinal epithelium and formation of lipid droplets. In this study, the increased abundance of *Bacillus* was found in the B3 and B5 groups, suggesting that *B. velezensis* YFI-E109 in the diet may cause its colonization in the gut of hybrid yellow catfish. When supplementation of *B. velezensis* YFI-E109 at a dosage of 0.83 × 10^12^ CFU/kg was provided, a dramatic rise in relative abundance of *Plesiomonas* was observed in the fish intestinal tract, which may have caused diarrhea. Therefore, dietary *B. velezensis* YFI-E109 supplementation at an appropriate concentration could improve the gut microbial structure in hybrid yellow catfish.

The liver is a key organ for controlling energy metabolism, and decomposition of amino acids to provide energy or for the synthesis of protein, glucose, and/or other biologically active molecules (Rui, 2014). Here, OPLS-DA showed highly significant differences between the group B0 and the groups fed with 0.85 × 10^10^ and 0.83 × 10^12^ CFU/kg *B. velezensis* YFI-E109 (B3 and B5). These metabolic differences are enriched in energy metabolism and amino acid metabolic pathways, suggesting that dietary supplementation with *B. velezensis* YFI-E109 has great influence on energy metabolism and amino acid metabolism in this fish species. In comparison with group B0, the relative levels of glucose, galactose, alpha ketone glutaric acid, succinic acid, and creatine phosphate were significantly affected in groups B3 and B5. Alpha ketone glutaric acid, succinic acid and fumaric acid are the intermediates of the TCA cycle, and the dehydrogenation of succinic acid is to produce fumaric acid for respiratory metabolism of H^+^ (Yang et al., 2018). In the present study, the decreased contents of UDP glucose and UDP-D-galactose with increased contents of fumaric acid and succinic acid were found in both group B3 and B5. These results indicated that adding *B. velezensis* YFI-E109 in the diet can promote glucose metabolism and TCA cycle activity in hybrid yellow catfish. Creatine phosphate is a high-energy phosphate compound in muscle or other excitatory organs (such as the brain and nerves). The elevated content of creatine phosphate in both groups B3 and B5 suggested that dietary *B. velezensis* YFI-E109 supplementation can contribute to the energy storage and transformation in fish. The result was similar to the findings in *Hermetia illucens* (Pei et al., 2022). However, in B3 vs. B5, most of the metabolites that displayed upward trend were decreased, indicating that whilst supplementing *B. velezensis* YFI-E109 at 0.85 × 10^10^ CFU/kg can promote nutrient decomposition in the liver and improve the growth of hybrid yellow catfish, high dose supplementation at 0.83 × 10^12^ CFU/kg could weaken beneficial effects. H&E staining also demonstrated that the fish in group B3 had less severe liver lesions than those in group B0.

## 5 Conclusion

In conclusion, dietary supplementation of *B. velezensis* YFI-E109 improved the growth, metabolism and intestinal health of hybrid yellow catfish. However, excessive supplementation of *B. velezensis* YFI-E109 could reduce growth performance and have negative effects on health. Based on the data of this experiment, the optimal supplemental dosage of *B. velezensis* YFI-E109 in the diet is approximately 0.31 × 10^10^ to 0.77 × 10^11^ for hybrid yellow catfish.

## Author contributions

**Zhehui Ji**: Feeding management, Formal analysis, Writing – original draft; **Xing Lu**: Writing – original draft, Writing - review & editing; **Mingyang Xue**: Screening and culture of the bacterial strains; **Yuding Fan**: Screening and culture of the bacterial strains; **Juan Tian**: Methodology; **Lixue Dong**: Methodology; **Chuanzhong Zhu**: Conceptualization, Funding acquisition; **Hua Wen**: Project administration, Funding acquisition, Resources, Supervision; **Ming Jiang**: Funding acquisition, Conceptualization, Project administration, Data curation, Writing - review & editing.

## Data availability statement

All data are available from the corresponding author by request.

## Declaration of competing interest

We declare that we have no financial and personal relationships with other people or organizations that can inappropriately influence our work, and there is no professional or other personal interest of any nature or kind in any product, service and/or company that could be construed as influencing the content of this paper.
