# Supplementary material for: The probiotic effects of host-associated Bacillus velezensis in diets for hybrid yellow catfish (Pelteobagrus fulvidraco ♀ × Pelteobagrus vachelli ♂)
Source: Anim Nutr. 2023 Aug 22;15:114–25. doi: 10.1016/j.aninu.2023.08.004 (PMC10665805; doi:10.1016/j.aninu.2023.08.004)
Supplement: Multimedia component 1 [file mmc1.docx]

**Supplementary Tables & Figures**

Table S1 Intestinal morphology measurements of hybrid yellow catfish fed diets supplemented with different levels of *Bacillus velezensis* YFI-E109 for 6 weeks^1^.

| Item | B0 | B1 | B2 | B3 | B4 | B5 | ANOVA-*P* |
| --- | --- | --- | --- | --- | --- | --- | --- |
| Diameter, µm | 847.32 ± 81.28 | 847.64 ± 15.042 | 848.13 ± 42.373 | 832.93 ± 20.394 | 819.28 ± 49.297 | 858.08 ± 16.183 | 0.991 |
| Villus height, µm | 238.92 ± 27.742 | 214.16 ± 12.108 | 223.79 ±50.726 | 213.57 ± 22.900 | 187.67 ± 12.463 | 243.19 ± 19.339 | 0.745 |
| Villus width, µm | 98.21 ± 9.130 | 92.94±1.823 | 113.72 ± 13.013 | 84.68 ± 7.056 | 73.19 ± 4.756 | 97.37 ± 7.143 | 0.057 |
| Muscularis thickness, µm | 89.29 ± 11.068 | 111.81±2.578 | 107.95 ± 16.126 | 117.42 ± 7.581 | 103.50 ± 4.571 | 166.57 ± 31.024 | 0.058 |
| Number of fluffs, pcs | 11.11 ± 1.444 | 11.33 ± 0.667 | 8.67 ± 2.404 | 9.89 ± 0.29 | 12.44 ± 1.056 | 11.50 ± 0.500 | 0.409 |

Data are presented as mean ± SE, *n* = 3.

Means in the same line sharing the same superscript letter are not significantly different, as determined by Tukey’s test (*P* > 0.05).

^1^The diets B0, B1, B2, B3, B4 and B5 contained 0, 0.90 × 10^8^, 0.80×10^9^, 0.85 × 10^10^, 0.90 × 10^11^, 0.83 × 10^12^ CFU/kg *Bacillus velezensis* YFI-E109, respectively

**Table S2** The number of sequences analyzed, estimated OTU richness (Species and Chao), and diversity index (Shannon and Simpson) for 16S rRNA libraries of mid-gut in hybrid yellow catfish fed diets supplemented with different levels of *Bacillus velezensis* YFI-E109 for 6 weeks^1^.

| Item | B0 | B3 | B5 | ANOVA-*P* |
| --- | --- | --- | --- | --- |
| Shannon | 1.63 ± 0.878 | 1.59 ± 0.573 | 1.49 ± 0.293 | 0.765 |
| Chao | 166.12 ± 99.051 | 71.33 ± 27.668 | 102.13 ± 4.645 | 0.472 |
| ACE | 159.78 ± 89.464 | 71.60 ± 27.597 | 134.23 ± 32.550 | 0.522 |
| Simpson | 0.52 ± 0.203 | 0.47 ± 0.196 | 0.34 ± 0.087 | 0.698 |

Data are presented mean ± SE, *n* = 3.

Means in the same line sharing the same superscript letter are not significantly different, as determined by Tukey’s test (*P* > 0.05)

^1^The diets B0, B3, and B5 contained 0, 0.85 × 10^10^, 0.83 × 10^12^ CFU/kg *Bacillus velezensis* YFI-E109, respectively.

**Table S3** Two comparisons of the major differential metabolites of hybrid yellow catfish fed diets supplemented with different levels of *Bacillus velezensis* YFI-E109 for 6 weeks (the FC value as the standard and *P* < 0.05)^1^.

| Item | Compared groups | T_*P* value | Fold change | PLS-DA-VIP | Status |
| --- | --- | --- | --- | --- | --- |
| 3,4-Dimethylbenzoic acid | B3_vs_B0 | 0.010 | 2.769 | 2.126 | up |
| N-(1-Deoxy-1-fructosyl) phenylalanine | B3_vs_B0 | 0.036 | 2.565 | 1.810 | up |
| 5-Aminovaleric acid | B3_vs_B0 | 0.009 | 2.252 | 2.120 | up |
| Cyclo (Phe-Glu) | B3_vs_B0 | 0.035 | 1.917 | 1.794 | up |
| N(α)-Acetyl-Epsilon-(2-Propenal) Lys | B3_vs_B0 | 0.033 | 1.742 | 1.738 | up |
| Creatine phosphate | B3_vs_B0 | 0.030 | 1.661 | 2.014 | up |
| Gly-Lys | B3_vs_B0 | 0.015 | 1.575 | 1.928 | up |
| Pregnanetriol | B3_vs_B0 | 0.002 | 1.551 | 2.203 | up |
| Lysophosphatidylglycerol (18:1/0:0) | B3_vs_B0 | 0.033 | 1.500 | 1.903 | up |
| Dehydroascorbic acid | B3_vs_B0 | 0.007 | 0.476 | 2.109 | down |
| L-Dopa | B3_vs_B0 | 0.036 | 0.472 | 1.920 | down |
| Nicotinamide riboside | B3_vs_B0 | 0.030 | 0.468 | 1.904 | down |
| Ubiquinone-1 | B3_vs_B0 | 0.035 | 0.460 | 1.830 | down |
| Ureidosuccinic acid | B3_vs_B0 | 0.004 | 0.455 | 2.266 | down |
| N-acetyl-L-histidine | B3_vs_B0 | 0.019 | 0.431 | 1.904 | down |
| Trigonelline | B3_vs_B0 | 0.007 | 0.276 | 2.137 | down |
| N-acetyl-L-glutamic acid | B3_vs_B0 | 0.049 | 0.224 | 1.864 | down |
| UDP-glucose | B3_vs_B0 | 0.027 | 0.214 | 2.061 | down |
| UDP-D-galactose | B3_vs_B0 | 0.027 | 0.214 | 2.061 | down |
| Succinic acid | B5_vs_B0 | 0.001 | 6.557 | 2.125 | up |
| Fumaric acid | B5_vs_B0 | 0.005 | 4.353 | 1.996 | up |
| Ritalinic acid | B5_vs_B0 | 0.000 | 3.249 | 2.311 | up |
| Ile-Trp | B5_vs_B0 | 0.031 | 2.923 | 1.608 | up |
| L-methionine sulfoxide | B5_vs_B0 | 0.000 | 2.852 | 2.192 | up |
| Creatine phosphate | B5_vs_B0 | 0.002 | 2.735 | 1.963 | up |
| γ-Tocopherol Metabolite | B5_vs_B0 | 0.032 | 2.376 | 1.673 | up |
| 4-Guanidinobutyric acid | B5_vs_B0 | 0.022 | 2.322 | 1.784 | up |
| Adipic acid | B5_vs_B0 | 0.004 | 2.112 | 1.934 | up |
| 3,4-Dimethylbenzoic acid | B5_vs_B0 | 0.044 | 2.093 | 1.454 | up |
| Gamma-mercholic acid | B5_vs_B0 | 0.010 | 0.365 | 1.799 | down |
| ST-638 | B5_vs_B0 | 0.006 | 0.360 | 1.962 | down |
| Azelaic acid | B5_vs_B0 | 0.035 | 0.342 | 1.618 | down |
| Triethylenetetramine | B5_vs_B0 | 0.000 | 0.339 | 2.304 | down |
| 8,8a-Deoxyoleandolide | B5_vs_B0 | 0.005 | 0.337 | 1.985 | down |
| UDP-glucose | B5_vs_B0 | 0.034 | 0.269 | 1.627 | down |
| UDP-D-galactose | B5_vs_B0 | 0.034 | 0.269 | 1.627 | down |
| α-Ketoglutaric acid（α-KG） | B5_vs_B0 | 0.001 | 0.253 | 2.143 | down |
| N-acetyl-L-glutamic acid | B5_vs_B0 | 0.043 | 0.192 | 1.548 | down |
| Gly-Thr | B5_vs_B0 | 0.007 | 0.000 | 2.009 | down |

FC = fold change; UDP = uridine diphosphate.

^1^The diets B0, B3, and B5 contained 0, 0.85 × 10^10^, 0.83 × 10^12^ CFU/kg *Bacillus velezensis* YFI-E109, respectively.


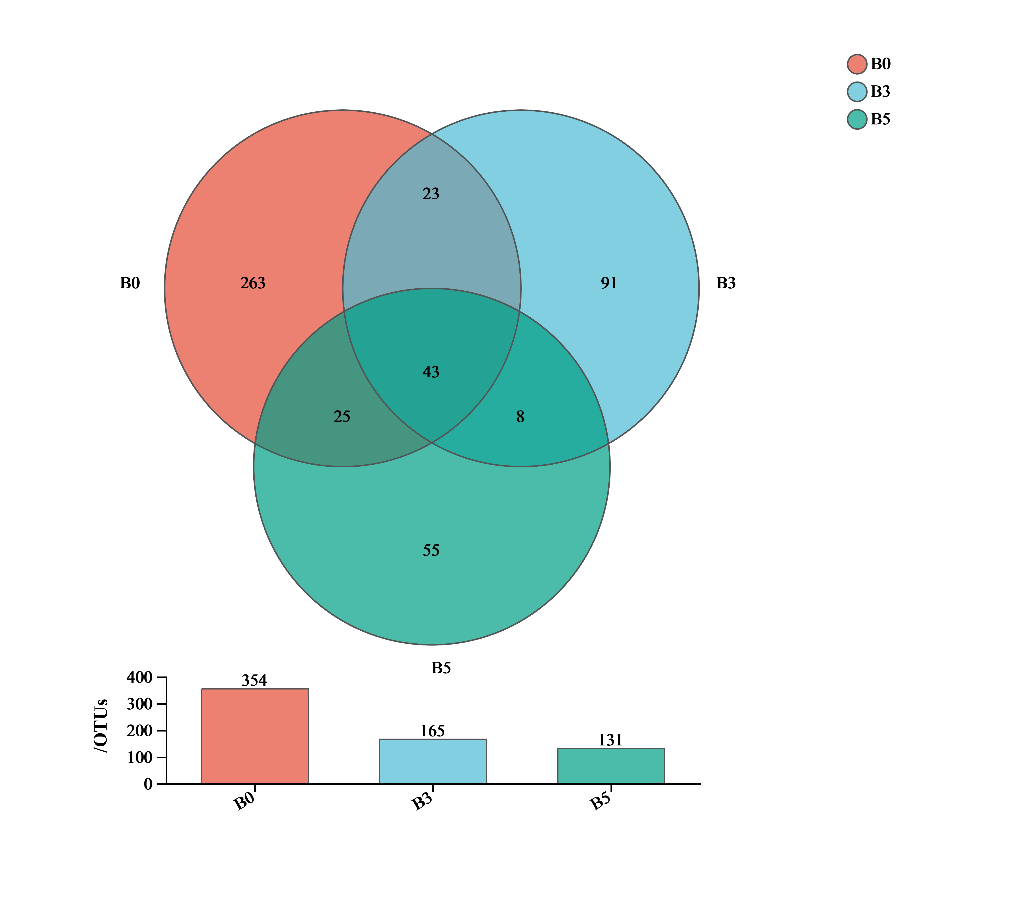


**Fig. S1** Venn diagram demonstrating the distribution of operational taxonomic units (OTUs) shared by yellow catfish fed with test diets. The diets B0, B3, and B5 contained 0, 0.85 × 10^10^, 0.83 × 10^12^ CFU/kg *Bacillus velezensis* YFI-E109, respectively.


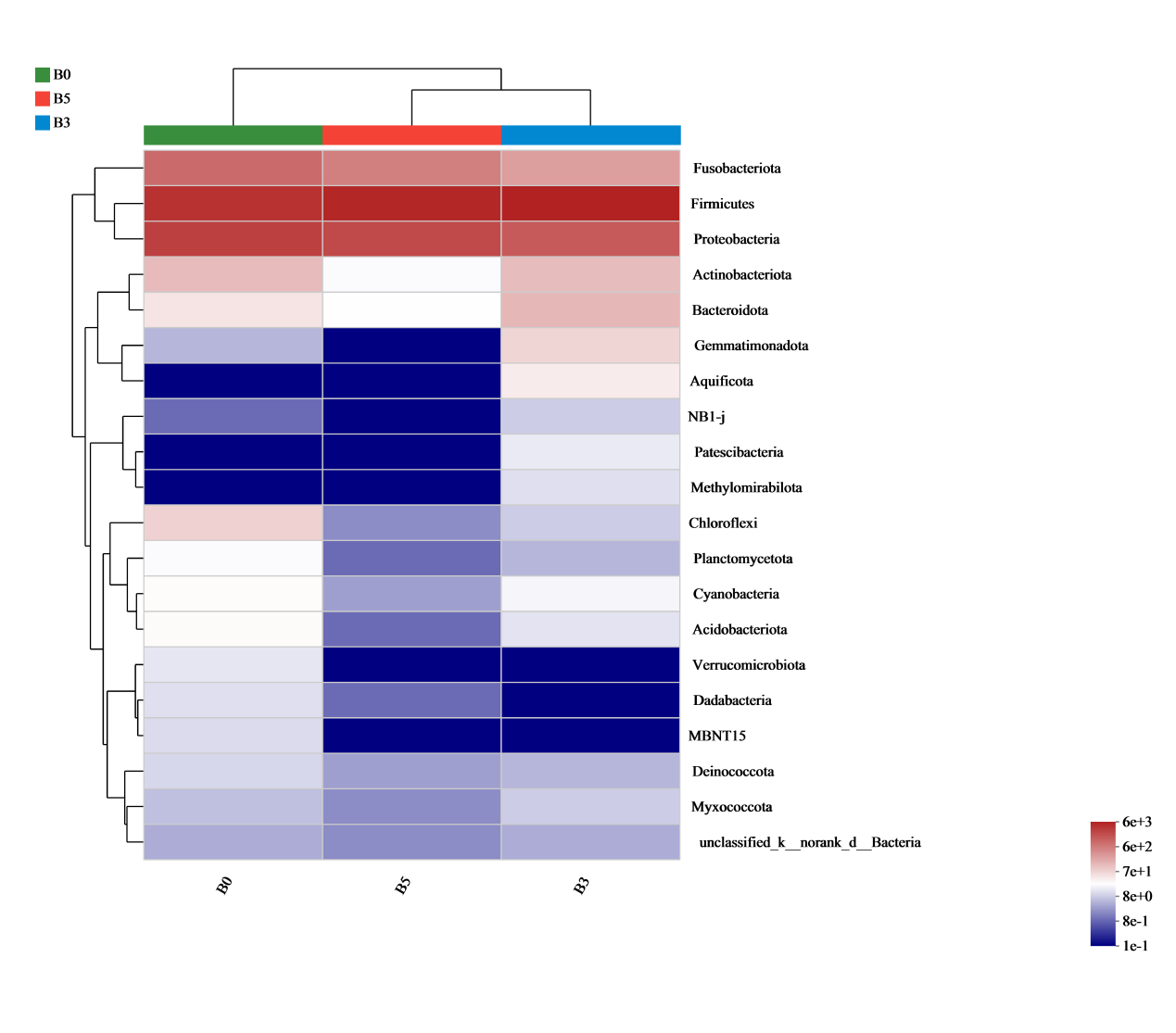


**Fig. S2** Community heatmap analysis of phylum level in yellow catfish fed with test diets. The diets B0, B3, and B5 contained 0, 0.85 × 10^10^, 0.83 × 10^12^ CFU/kg *Bacillus velezensis* YFI-E109, respectively.


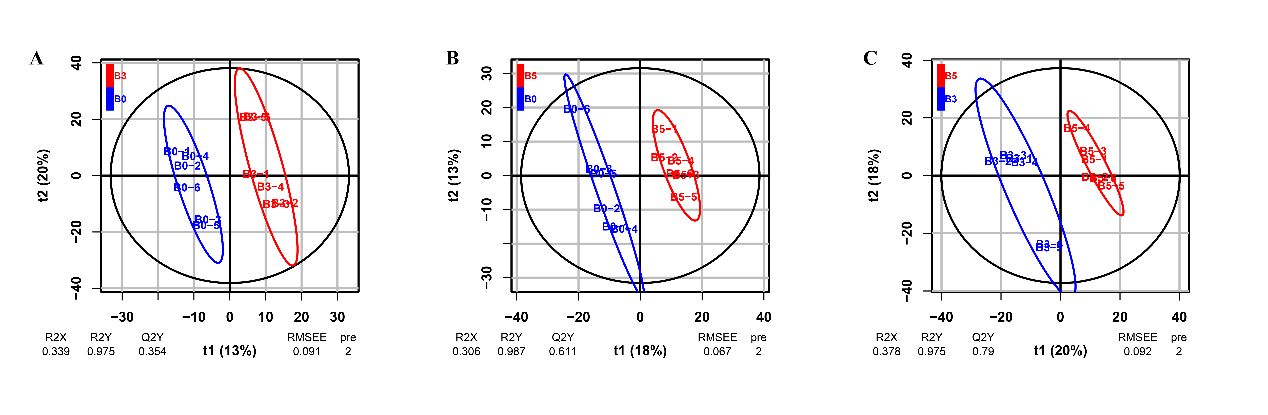


**Fig. S3** PLS-DA scores for the differential metabolites of the hybrid yellow catfish B0, B3 and B5 groups based on integration mode. (A) B3 vs B0; (B) B5 vs B0; (C) B5 vs B3. The diets B0, B3, and B5 contained 0, 0.85 × 10^10^, 0.83 × 10^12^ CFU/kg *Bacillus velezensis* YFI-E109, respectively.


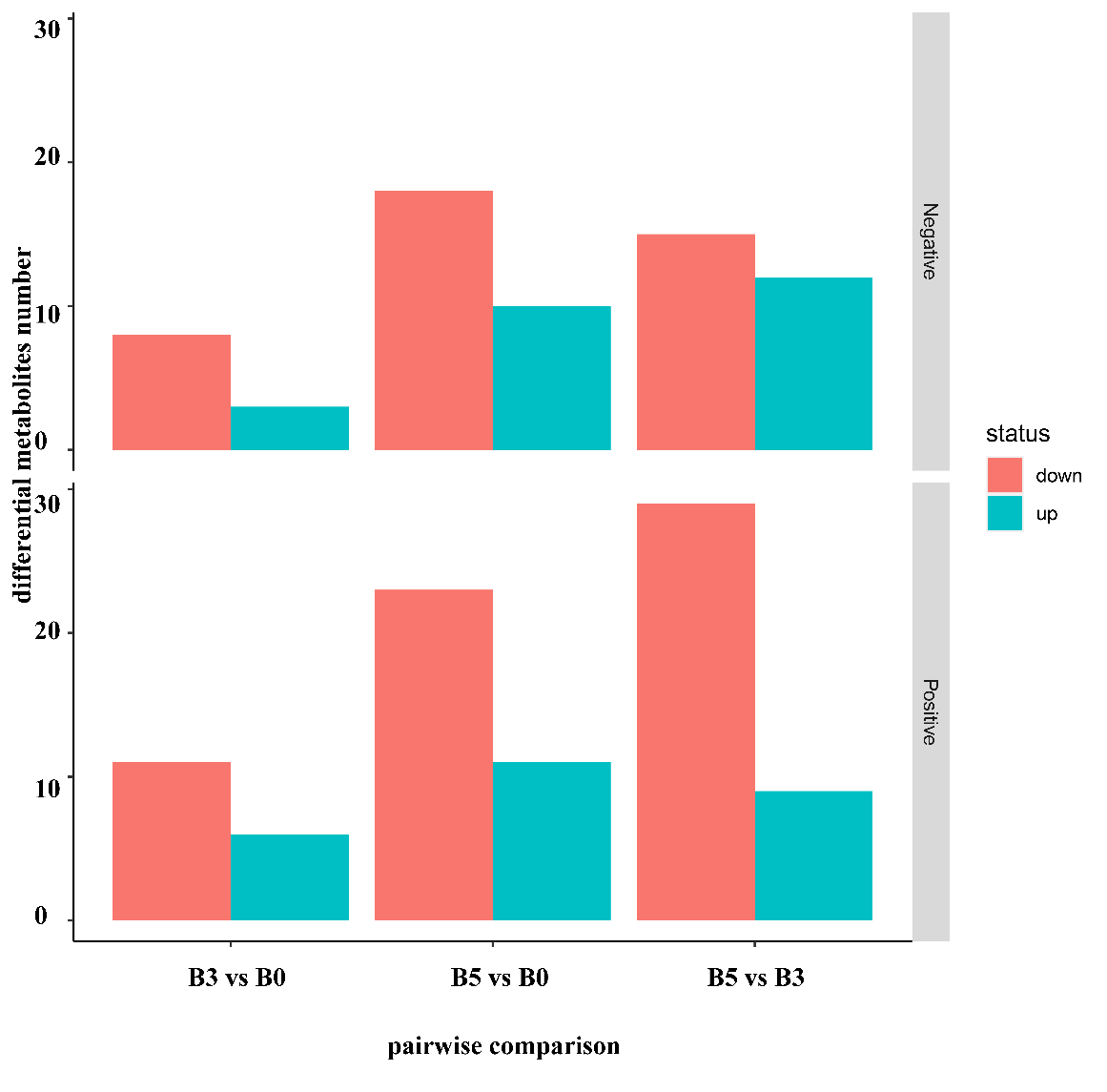


**Fig. S4** Bar plot of differential metabolite number statistics (according to their VIP > 1 and *P* < 0.05 values). The diets B0, B3, and B5 contained 0, 0.85 × 10^10^, 0.83 × 10^12^ CFU/kg *Bacillus velezensis* YFI-E109, respectively.
